# Repercussions of imprisonment for conjugal violence: discourses of men**^1^**

**DOI:** 10.1590/1518-8345.1569.2847

**Published:** 2016-12-08

**Authors:** Anderson Reis de Sousa, Álvaro Pereira, Gilvânia Patrícia do Nascimento Paixão, Nadirlene Gomes Pereira, Luana Moura Campos, Telmara Menezes Couto

**Affiliations:** 2 MSc, Professor, Universidade Federal do Recôncavo da Bahia, Santo Antônio de Jesus, BA, Brazil.; 3 PhD, Associated Professor, Escola de Enfermagem, Universidade Federal da Bahia, Salvador, BA, Brazil.; 4 Doctoral student, Escola de Enfermagem, Universidade Federal da Bahia, Salvador, BA, Brasil.; 5 PhD, Adjunct Professor, Escola de Enfermagem, Universidade Federal da Bahia, Salvador, BA, Brazil.; 6 Master's student, Escola de Enfermagem, Universidade Federal da Bahia, Salvador, BA, Brazil.; 7 PhD, Professor, Escola de Enfermagem, Universidade Federal da Bahia, Salvador, BA, Brazil.

**Keywords:** Gender Identity, Masculinity, Men's Health, Violence Against Women, Nursing

## Abstract

**Objective:**

to know the consequences that men experience related to incarceration by conjugal violence.

**Methods:**

qualitative study on 20 men in jail and indicted in criminal processes related to conjugal violence in a Court specialized in Family and Domestic Violence against women. The interviews were classified based on Collective Subject Discourse method, using NVIVO^(r)^ software.

**Results:**

the collective discourse shows that the experience of preventive imprisonment starts a process of family dismantling, social stigma, financial hardship and psycho-emotional symptoms such as phobia, depression, hypertension, and headaches.

**Conclusion:**

due to the physical, mental and social consequences of the conjugal violence-related imprisonment experience, it is urgent to look carefully into the somatization process as well as to the prevention strategies regarding this process.

## Introduction

Conjugal Violence, an alarming public health problem, is defined as any action or omission against the person with whom the aggressor has an emotional involvement. It is anchored usually in an asymmetric relationship guided by the inequality between the partners. From a gender perspective, masculinity and femininity are anchored in the social construction of what is "being a man" and "being a woman", so that we all learn, naturalize and reproduce the expected roles for each sex^1^. This symbolic belief guides the formation of identities guided by the male hegemony, that portraits women as inevitably inferior or subservient to men, thus inciting violence against women^2^.

Domestic violence has been the subject of many studies around the world, both for its various forms of expression, as for the consequences for those involved, being considered a trans-generational and interactional phenomenon^3^. In spite of the existence of mutual aggression, there are specificities in the form of attack, as well as in the repercussions when observed under a gender approach^4^. Women often employ light physical aggression, such as scratches, as well as verbal and psychological violence; men due to superior physical strength, tend to cause severe lesions and therefore their violence have a higher profile^5^. For this reason, although studies agree about the reciprocal nature of domestic violence there is an emphasis on the damage produced on the health of women^6^ ^-^ ^7^.

However, some sparse research points to the existence of male repercussions. One example is a study conducted in the United States, whose results showed that men engaged in love relationships permeated by violence have higher rates of psychiatric problems, such as moderate or severe depression and post-traumatic disorders^8^. These effects are intensified with the experience of incarceration, where individuals are faced with shortage of food and hygiene products; lack of adequate infrastructure; violence among inmates, as well as trafficking and use of illicit drugs^9^.

Despite the scarce production of knowledge on consequences of domestic violence for the male population, we remark the importance of identifying health and care needs of this population. Therefore, it is urgent the inclusion of man author of domestic violence, as central subject of studies. Given this gap in the literature the following research question is proposed: what are the consequences of serving time for domestic violence for men? The objective adopted was: to know the repercussions in men of imprisonment for domestic violence.

## Methods

This is a descriptive, qualitative study, linked to a larger project funded by *Fundação de Amparo à Pesquisa do Estado da Bahia* - FAPESB (Research Foundation of the state of Bahia, Brazil) entitled: Re-education of men and women involved in criminal processes: the domestic violence coping strategy. This project is developed by the Study Group on Violence and Quality of Life belonging to the Nursing School of the Federal University of Bahia. It aims to create a gender-oriented social technology for men and women re-education, through the development of reflection groups (RG).

The participants were 20 men indicted for domestic violence, previously arrested, and responding in probation at the time of the study, to criminal prosecution in a Court specialized in Family and Domestic Violence against women in the city of Salvador, Bahia, Brazil. The selection of participants was intentional, and the approach strategy was to invite them to participate in RG meetings. The Court's Social Service made the first contact when potential participants went to the institution to attend their hearings of the case. Researchers contacted by telephone those individuals who were interested and gave authorization to participate, stating the date to integrate the group.

The outlined inclusion criteria were: men indicted in criminal proceedings for domestic violence in that specific Court after experiencing temporary incarceration. We excluded men with processes whose cause was based on other type (non-conjugal) of violence against women.

On the first day of the Reflective Group, participants were informed about the purpose and relevance of research; potential benefits and risks; right to decide whether to collaborate or not with the research, ensuring that the refusal would not result in exiting the group, and other ethical principles recommended by the National Health Council Resolution 466/12. Those accepting to participate in the study signed a free and informed consent form and scheduled a time of their convenience to be interviewed. The Ethics Committee of the Federal University of Bahia approved the project under number 877,905.

Data collection took place between the months of June to October 2015, through individual interviews, lasting an average of half an hour. The semi-structured interview was guided by the question: what are the imprisonment repercussions for your life? The research team was composed of nurses and nursing researchers undergoing master's and doctoral courses, under the supervision of PhDs with extensive experience in this area of knowledge. The speeches of interviewees were recorded, transcribed and identified by the letter H and the number of realization of order, such as H1, H2 and beyond. We did not consider the risk of bias for joining the RG, since the interview referred to the repercussions of the prison experience, that was the topic of the group until the completion of data collection. To maintain rigor in the study, the interviews were made available after the transcript to all participants, in order to check whether they were represented in the way the data were transcribed. For such a strategy, as a support tool, we met the Consolidated Criteria for Reporting Qualitative Research (COREQ). The initial organization of the transcribed material was performed using the *software* NVIVO^(r)^ version 11 without any financial contribution, followed by categorization of core ideas and construction of syntheses speeches through the Collective Subject Discourse method. Based on the studied material, it was elaborated a "word cloud", representing the frequency of evoked words, highlighting the most frequent words in the speech. This is showed in the results, and supports the findings, which were based from the themes: violence, gender and masculinities.

## Results

### Central Idea 1 - Consequences for health

In the evaluation of the speeches about the repercussions of being imprisoned for domestic violence, it was possible to see that they point to the somatization of the experience, illustrated through the following subcategories: mental illness and physical illness.

### Central synthetic idea 1A - Mental Illness

The experience of being in jail associated with domestic violence is unveiled as an event that generates psycho-emotional effects, like the hypervigilance, sadness, low self-esteem, self-deprecation, apathy and depression, as shown in the following speech: *It shocked my brains. My conscience is heavy. In the first months, I was a shame, lying on the floor at home, in solitude. I feel empty, I feel useless, discouragement to work, to live. I feel sad and cry a lot. Self-esteem is down there. I became depressed. The head, in a matter of seconds, can lead you to make a mess. I've thought about leaving everything and disappear in the world. I feel upset, unbalanced, traumatized. I walk around scared. When passing a car on the street, I get nervous thinking it will get me. We live nearby, so I'm afraid to know that any time a person can call the police* (refers to restraining order). *I also have nightmares. All this makes me feel ill.* (DSC, H1, H2, H3, H4, H5, H6, H7, H8, H12, H13, H15, H18, H19, H20).

### Central synthetic idea 1B - Physical Illness

 Respondents' speeches also show the relationship between the experience of prison and the unleashing of physical signs and symptoms, such as gastric alterations; weight loss associated with loss of appetite; reduced muscle strength; changes in sleep patterns; headache; tachycardia and hypertension. *After the event, I lost appetite, lost quite a lot of weight. I think I got gastritis. I cannot pick up more weight. I cannot sleep, I am sleepless. The heart is also tight, I feel chest pain, and I'm short of breath. I started to have hypertension, feel dizziness, headache and I'm nervous. I am no longer the same man* (DSC H1, H2, H3, H5, H7, H8, H9, H10, H14, H17,H19, H20).

### Central Idea 2 - social repercussions 

This category includes repercussions on the socio-economic sphere that create vulnerability in men who were in prison after assaulting their companions. These can be illustrated from the following subcategories:

### Central synthetic idea 2A - Family Breakdown

 The speech shows that the men's incarceration generates alienation not only regarding his female partner, but other significant others as well, such as children, parents and siblings. It also expressed the vision of indispensability of the man-father at home, so that their removal is a risk of deviant behavior of the children. *It's cruel because now I am alone! Everybody's gone. I miss everybody! I totally moved away from my family. I lost my wife for no reason, we had a life project, we have already agreed to grow old together. She does not want to see me. Also, I have to stay away from my children because she prevents it. Homesickness beat and the heart was tight, because I knew he was missing me. And the justice forces me to stay 300 meters away, or I'll be arrested again. I am in bad shape! I think in their growth, because a father is the guide of the house. My children are living with serious problems* (silence, eyes filled with tears). *The boy is not going to school, the girl is struggling to study. I see that if we do not keep our children, they will find a way to throw themselves in this world.* (DSC, H1, H2, H3, H4, H5, H7, H9, H11, H13, H15, H17).

### Central synthetic idea 2B - Social stigma

When arrested for practicing violence against women, such male behavior becomes public. As a result, the study reveals that men become labeled and harassed by people in their social life, both in the community where they resides as in the professional sphere, including those that were part of their friendship circle. *The people in my community do not look at me like before. They even say: look at the man who likes to hit women. My former boss was saying, you're an ex-con. Sometimes I'm afraid to give my resume in business and they see that I am a former detainee. People say: "he is a criminal, he is an aggressor, he is guilty." Even in lectures that are out there, I'm considered the aggressor. I feel a defendant, and I think that this will never end. I feel humiliated, embarrassed, ashamed.* (DSC, H2, H5, H6, H9, H10, H13, H16, H18, H19, H20).

### Central synthetic idea 2C - Financial hardships

The prison has financial implications, considering that there are more difficulties in finding formal jobs after this event. Men express anger and suffering because they cannot afford food expenses, water, energy, aid for children and rent, due to alienation from home as disposed by the restraining order issued by the responsible judge. *I spent a month in jail and eight months unemployed, unable to work formally because I am indicted. I have picked in the garbage for food to eat. I sold a television to pay water bills, electricity, rent and other. Sometimes I'm wondering how it got this far if I had everything in my house? Moreover, it has harmed my daughters, because I have their things to pay, and I will not be able to afford, because I still have to pay the lawyer. Justice should split half and half the property, it is also mine, but I did not have that right.* (DSC, H1, H2, H4, H5, H7, H12, H14, H17).

**Figure 1:**
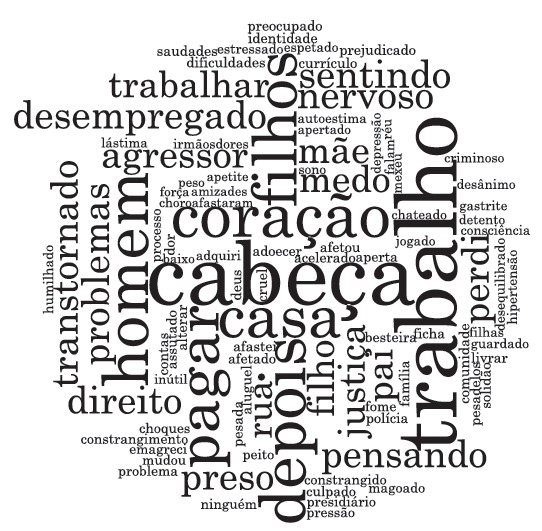
Word cloud generated by the *Software* NVIVO^(r)^ version 11 - Query of frequency of words appearing in the collective speech, 2015, Salvador, BA, Brazil

The consequences for men of the imprisonment for conjugal violence, emerging from the aforementioned categories, are backed in a "word cloud" (Figure 1) made up by the words that express the central ideas of this study.

## Discussion

The study points to the relationship between experiencing the prison as a consequence of domestic violence and developing mental illness, expressed by the phobia, low self-esteem, self-deprecation, depression, among others. This finding supports other research conducted with men confned in Minas Gerais, Brazil, whose findings reveal that prison can cause various diseases, especially stress and depression^10^, presenting itself as an important trigger of mental severe disorders^11^. These symptoms of psycho-emotional character without defined physiological cause, are related to the somatization process of the experience, transferring to the body the problems originated in the mental sphere.

The somatization of the jail experience in turn, is not restricted to mental illness, expressing itself also through clinical signs and symptoms, such as loss of appetite, gastrointestinal problems, hypertension and headache. These findings corroborate a study of men in prison in Rio Grande do Norte, Brazil, which has listed headache, diarrhea and weight loss as physical symptoms caused by the experience of imprisonment^11^.

International researchers also highlight the process of getting ill as suffered by men in the prison system, with the onset of varied physical and mental symptoms. To have an idea of the magnitude of this illness in the United States, in mid-2005 more than half of all inmates of an American prison had mental health problems with somatization of the physical type^12^, expressed in symptomatology convergent with those found in this study. 

One of the reasons given for this psychosomatic illness concerns the prison as a modifier environment of the way how a man acts, as it requires to have a passive, restrained and solitary behavior, which goes against what is the socially expected condition of 'being a man"^10^. From this perspective, the collective subject of this study reveals a sense of decreased muscle strength, with reports of personal and subjective changes when states "I am no longer the man I used to be". This condition seems to be related to the composition of gender and identity that is intrinsic with the variables physical strength and virility, expected from a man. This idea of what is to be a man is present in masculinity studies. A scholar on masculinities, Socrates Nolasco argues that "contemporary societies allow man to make use of physical force as a means to show virility"^13^. Thus, the decrease in muscle strength may be related to the perception of decreased male role, as if the experience in prison makes the person to be "less of a man", and this change is revealed as another symptom of somatization of the experience. 

The study also calls attention to feelings such as fear, which is revealed when the collective subject refers to the fear of a new complaint due to the breach of urgent restraining measures. This could be evidenced when it is mentioned the fact of living close by to the former partner as in the speech Central synthetic idea 1A. The restraining order is provided by Law 11.340 / 2006 (known as *Maria da Penha Law*) and summarizes behaviors that must be respected by men in order to ensure the protection of women and / or children, such as suspension or restriction of bearing weapons; keeping distance from domicile or place of living of the victim; prohibition of behaviors such as approach and contact with the woman, family and witnesses, while respecting the minimum distance; suspension and restriction of visits to dependent minors^4^. It is important to note that in cases where the woman wants a rapprochement with the man, considering that a criminal case is ongoing, the lawsuit will not be suspended. However, she can request the revocation of urgent restraining measures.

The discourses also call attention to the fact that the man does not understand the act occurred as severe and considers that the end of the relationship 'was without cause'. Studies show that, due to the naturalization of violence, men have difficulties to recognize themselves as perpetrators of violence and therefore they do not understand why are they being charged^14^ ^-^ ^15^. International researchers show that the way men see themselves, whether in their private, social, institutional and / or political relationships, condition them to exercise domination, control and violence against women, since there is an understanding of these acts as normal and typical of the male, which hinders the understanding of men that the acts committed by them constitute a form of violence^16^.

Inefficiency in the dissemination of policies regarding violence against women control policies, the wrongly interpretation of the law in police stations and in courts and the culture of impunity among male offenders may be contributing to hinder the social denaturalization of such violence and the understanding of the forms of violence that they practice in their conjugal relations.

The removal from children could also be shown as something that causes suffering to men. In this context, the speech refers to two situations in which there is separation between father and son. The first refers to the restraining orders that as already mentioned sometimes determines the suspension of visits to minor children, a conduct that is adopted whenever there is a consideration of integrity at risk. The second situation is when the woman prohibits or hinders this relationship, which characterizes parental alienation.

Marked by the abusive interference of one parent in the psychic conception of the offspring over the other non-guardian parent, parental alienation is a behavior commonly committed by women after separation, being described by authors as a means of retaliation toward their former partners^17^.

It is important to discuss this situation, since the separation can trigger the "parental alienation syndrome", that are the emotional and behavioral sequelae that appear almost exclusively in the context of child custody disputes. To prevent this event, the 12.318 / 2010 law was sanctioned in order to curb or reduce parental alienation in Brazil, thus presenting possible solutions to eradicate these acts harmful to the healthy growth of children and adolescents, especially through shared custody^18^.

In the speeches, besides demonstrating that men miss their children, the collective subject reveals a strong concern with their everyday life without men. Added to this there is a perception that parental influence is basic for keeping the house in order and for the development of the child. This concept is strongly related to the deep-rooted gender inequalities in society considering that such relationships are focused on the paternalistic view that bestows on men the centralizing power as breadwinners and home providers^8^.

In addition to the separation from children, the study reveals the estrangement from other family members. Research conducted in Colombia with jailed men, confirms that the incarceration process dramatically affects the composition of the family^19^. Considering that the alienation from family members is linked to the experience of prison, the study points out the social stigma of being a (former) inmate, and not exactly the social perception of domestic violence considered as crime. This is because usually the first recorded violence occurs years after theirs, so oftentimes it was already something the family knew well.

A Brazilian study regarding indicted men's conceptions about domestic violence shows that those cases occur in symbolic home environments that facilitate the husbands' power over their wives. Added to this context there is a popular proverb that states: "Nobody should intervene in a man and wife quarrel." To strengthen it, the study reveals that men reinforce their position as heads of the home and reaffirm the couple's privacy in the same way that protect themselves from the convictions involved in this process, even when they are on the public knowledge^20^. This situation is also supported by the women's position of subservience, based on models of family and conjugality.

The female empowerment for a life free of violence often involves the decision to complain, which often culminates in the imprisonment situation. This is an event that has a strong association with the establishment of social "labels"^19^, making men to feel embarrassed, humiliated and ashamed of what has happened^21^. 

Concerning the economic aspects, the study shows the interface between the stigma of jail and the dismissal from employment, an event that impacts creating financial difficulties, as reported in the collective discourse. Corroborating these findings, a Brazilian research in correctional settings unveils the clumsiness feeling facing everyday problems, in addition to having a restriction on their social and economic resources^10^. This reality of social exclusion was also evident in a study conducted in Israel, in which former detainees have difficulties in returning to the activities performed previously, including their jobs^22^. In this sense, and due to the stigma in addition to the isolation from family members, men can have difficulties in paying their basic daily expenses. This situation is worsened by the fact of having to bear extra costs, such as attorneys and rent. It is noteworthy that the removal from the house is due to urgent restraining orders derived from what happened, but it does not deny the men's right to have part of the assets acquired in the marital relationship. Therefore, there is a need of another legal process related to the sharing of common property.

Due to financial conditions, men also are concerned in not being able to meet their financial responsibilities to their children and therefore there are some indispensable reflections on this subject. Thus, even assuming the clear perception of criminal acts to women, scholars believe that the prison has not been the best strategy for rehabilitation and / or recovery of men. This process is not even closer to the impacts it aims to achieve, because the labor and educational activities in the Brazilian penitentiary environment, are generally poor^23^. After serving their time, is hard for these men to re-enter to their 'old' social and professional environment. This puts them in a position of lack of change, or even taking a violent way of life. The experience has increased intolerance and strengthened stigma because people that served time in the prison system are usually abhorred by society^21^.

It is necessary to stress the importance of re-socialization and reintegration of men in the work process after the prison experience, requiring the mobilization of various social sectors, including the family^24^. In this context, experiments carried out in Australia have sought effective strategies to prevent violence and contribute to the rehabilitation of men in prison, to society and work, by using a variety of pedagogic approaches, involving the participation of teachers, caregivers, health professionals, community leaders and public figures, in order to increase their safety, development of equitable attitudes and to engage them to promote healthy relationships. Efforts also include incentives to parenting programs, education in relationships, policies and programs for families^25^.

## Conclusion

Indicted men's discourse emerges that the prison by marital violence experience triggers mental and physical illness, which is the result of the somatization experienced. In addition, it causes family breakdown, raises stigma and causes financial difficulties. Despite the need of penalizing male perpetrators regarding their violence against women, it is essential to think about strategies that minimize the potential risk of illness for the male population due to the prison experience. Faced with the limited production of knowledge about the subject in question, the findings draw attention to the harm, albeit indirect, that the experience of domestic violence impinges on the health and employability of men. It points out the need for further research in search of theoretical studies that support intervention on the issue, considering its potential damage to the whole family. 

Considering being a man and being a woman as social constructions, it must be assumed that since childhood we are taught the proper behavior for each sex in order to naturalize male dominance and female subservience. Faced with the above, as relevant as the punitive measures are the actions in order to prevent violence against women, focusing on the promotion of gender equality. These actions should be coordinated with the Family Health Strategy and can be carried out, for example, with families in the community; with school children and adolescents; or even preferably with those companies that have functional concentration of a particular gender, such as sewing and transport, groups with women or men. The aim is to facilitate the construction of more symmetrical models between genders, no longer guided by the submission of one another, halting in this way the perpetuation of violence against women in the following generations.
